# Predicting drug−disease associations via sigmoid kernel-based convolutional neural networks

**DOI:** 10.1186/s12967-019-2127-5

**Published:** 2019-11-20

**Authors:** Han-Jing Jiang, Zhu-Hong You, Yu-An Huang

**Affiliations:** 1 Xinjiang Technical Institute of Physics and Chemistry, Chinese Academy of Science, Ürümqi, 830011 China; 2 University of Chinese Academy of Sciences, Beijing, 100049 China; 3 Xinjiang Laboratory of Minority Speech and Language Information Processing, Urumqi, China; 4 Department of Computing, Hong Kong Polytechnic University, HungHom, Hong Kong

**Keywords:** Sigmoid kernel, Convolutional Neural Networks, Random forest

## Abstract

**Background:**

In the process of drug development, computational drug repositioning is effective and resource-saving with regards to its important functions on identifying new drug–disease associations. Recent years have witnessed a great progression in the field of data mining with the advent of deep learning. An increasing number of deep learning-based techniques have been proposed to develop computational tools in bioinformatics.

**Methods:**

Along this promising direction, we here propose a drug repositioning computational method combining the techniques of Sigmoid Kernel and Convolutional Neural Network (SKCNN) which is able to learn new features effectively representing drug–disease associations via its hidden layers. Specifically, we first construct similarity metric of drugs using drug sigmoid similarity and drug structural similarity, and that of disease using disease sigmoid similarity and disease semantic similarity. Based on the combined similarities of drugs and diseases, we then use SKCNN to learn hidden representations for each drug-disease pair whose labels are finally predicted by a classifier based on random forest.

**Results:**

A series of experiments were implemented for performance evaluation and their results show that the proposed SKCNN improves the prediction accuracy compared with other state-of-the-art approaches. Case studies of two selected disease are also conducted through which we prove the superior performance of our method in terms of the actual discovery of potential drug indications.

**Conclusion:**

The aim of this study was to establish an effective predictive model for finding new drug–disease associations. These experimental results show that SKCNN can effectively predict the association between drugs and diseases.

## Background

New drug discovery is expensive due to the increasing challenges in drug target identification and drug design. Drug development normally contains three phases: the discovery phase, the preclinical phase, and the clinical development phase, each of which cost a lot of time and money. Nowadays, developing new drug generally takes 13–15 years and costs an average of $2 billion to $3 billion, which is continuing to increase. As the efficacy and side effects of older drugs are still not fully understood, there is growing interest in using older drugs to treat other diseases for which they were not originally designed. Some redirected drugs have been successfully identified by casual or rational observations. In view of this, it is an urgent need to utilize an efficient and scalable approach for identifying the associations between old drugs and disease on a large scale.

In recent years, a large number of computational methods have been proposed to predict drug–disease associations. For instance, Chen et al. proposed a method called HNBI, which is based on an allogeneic network for drug indication prediction [1]. However, drug repositioning applying this method requires drug target-miRNA and miRNA-disease associations, which is limited in number. Chandrasekaran et al. proposed to apply and combine multi-perspective and multi-approach learning to study the association between drugs and diseases [2]. However, the approach they propose needs to incorporate a lot of multi-source information. Huang et al. used a network communication method to integrate drug–protein interaction networks and use gene expression profiles to infer and assess the probability of drug and disease occurrence [3]. However, the application of this method is limited due to its need for the expression profile of target genes as input data, which, in most cases, is unavailable. Luo et al. proposed a recommendation system called DRRS [4]. They predict new drug indications by integrating data sources and validation information relevant to drugs and diseases. The effectiveness of DRRS could be negatively affected by the sparsity and similarity measurement of data sets that they use.

As the materials for classification problem in data mining, raw data contain useful information that is benefit for prediction performance as well as large noise information, which poses the major challenge for the prediction task [5]. Feature extraction is proposed to learn the most meaningful features for each sample, discarding the noise from the raw data. It is an important area in conventional researches in bioinformatics, especially for those associated with drugs. For example, Liang et al. extracted characteristics from LRSSL by combining molecular fingerprints of drugs and extracting important drug characteristics from multiple drug characteristic spectra under the constraint of L1-norm [6]. Zhang et al. propose the model of SCMFDD applying a matrix containing only 0 and 1 to represent features, which can only represent the existence of substructures, targets, or drug interactions [7]. The calculation of drug repositioning can also consider the application of deep learning to extract features.

In recent years, deep learning methods have made remarkable progress in solving such problems as natural language processing, image recognition and speech recognition [8]. It proves to be effective in solving different types of problems in data mining, opening a new avenue for the application of bioinformatic tools. Thus, the application of deep learning in feature extraction for drugs is gaining increasing attention [9]. For example, DeepCCI proposed by Kwon et al. uses a Convolutional Neural Network to automatically extract the Simplified Molecular Input Line Entry Specification (SMILE) features of chemicals [10]. Along this promising direction, this work proposes a novel feature extraction method based on Convolutional Neural Network (CNN) for learning a meaningful feature representation of drug–disease associations.

As supplement to clinical experiments for identifying drug–disease associations, computational methods based on statistic rules and machine learning are low-cost and fast [11]. In addition, they are able to integrate different types of data resources relevant to diseases and drugs and can therefore yield the most potential candidates for experimental validation. Much effort has been devoted towards this promising direction. For example, MBiRW uses a comprehensive similarity measure and a dual random walk algorithm to identify potential indications for a given drug [12]. DrugNet is method which is proposed based on a heterogeneous network of interconnected drugs, proteins, and diseases for testing different types of drug relocations [13]. HGBI is based on graph reasoning for achieving network drug and target correlation prediction [14]. Although HGBI is used to predict the association between drugs and targets, it is based on the prediction of drugs and diseases. KBMF is a combination of dimensionality reduction, similarity decomposition and binary classification to predict drug target interaction network [15]. DRRs proposed a drug repositioning recommendation system to predict new drug indications by integrating relevant data sources [4].

A large number of drug–disease associations have been confirmed by clinical studies and stored in some public databases. However, the number of such data is still limited for fully understanding the effects of drugs on diseases. In this study, we propose a computational method for drug repositioning which combines Sigmoid Kernel and Convolutional Neural Network (SKCNN). The SKCNN combines multiple sources of data information, including drug sigmoid kernel similarity, drug structural similarity, disease semantic similarity and diseases sigmoid kernel similarity. Specifically, in the first step of our methods, the drug structure similar network and the disease semantic similar network are combined with the sigmoid kernel network to obtain the drug and disease similar descriptors [16]. Secondly, the Convolutional Neural Network technology is used to extract the useful information of drug and disease similarity symbols for representing their interactions and further combine them as the final feature descriptor. Finally, the feature descriptor is used as the inputs of the random forest classifier to predict the association of each type of drug with all diseases.

To evaluate the performance of SKCNN, tenfold cross validation was implemented on the gold dataset. As a result, SKCNN obtained 91.65% prediction precision with 87.07% recall at the area under the curve (AUC) of 95.11%. In comparison with different classifier, SKCNN also achieved good results. In addition, we validated the proposed model against two human disease including obesity and asthma. As a result, more than 15 of the top-20 drug candidates (15/20 for obesity and 17/20 for asthma) predicted by SKCNN were successfully confirmed in comparative toxicogenomics database (CTD database) [17]. These experimental results indicated that SKCNN is effective to predict drug–disease associations on a large scale.

## Materials and methods

In this section, we introduce a novel drug repositioning computational method using Sigmoid Kernel and Convolutional Neural Network (SKCNN). In this section, we first give a brief description of the used datasets. Second, we explain how drug similarity and disease similarity are computed based on the known drug–disease association. Third, feature extraction based on the convolution neural network is explained. Finally, we show the experimental results yielded by random forest based on cross validation [18].

**Fig. 1:**
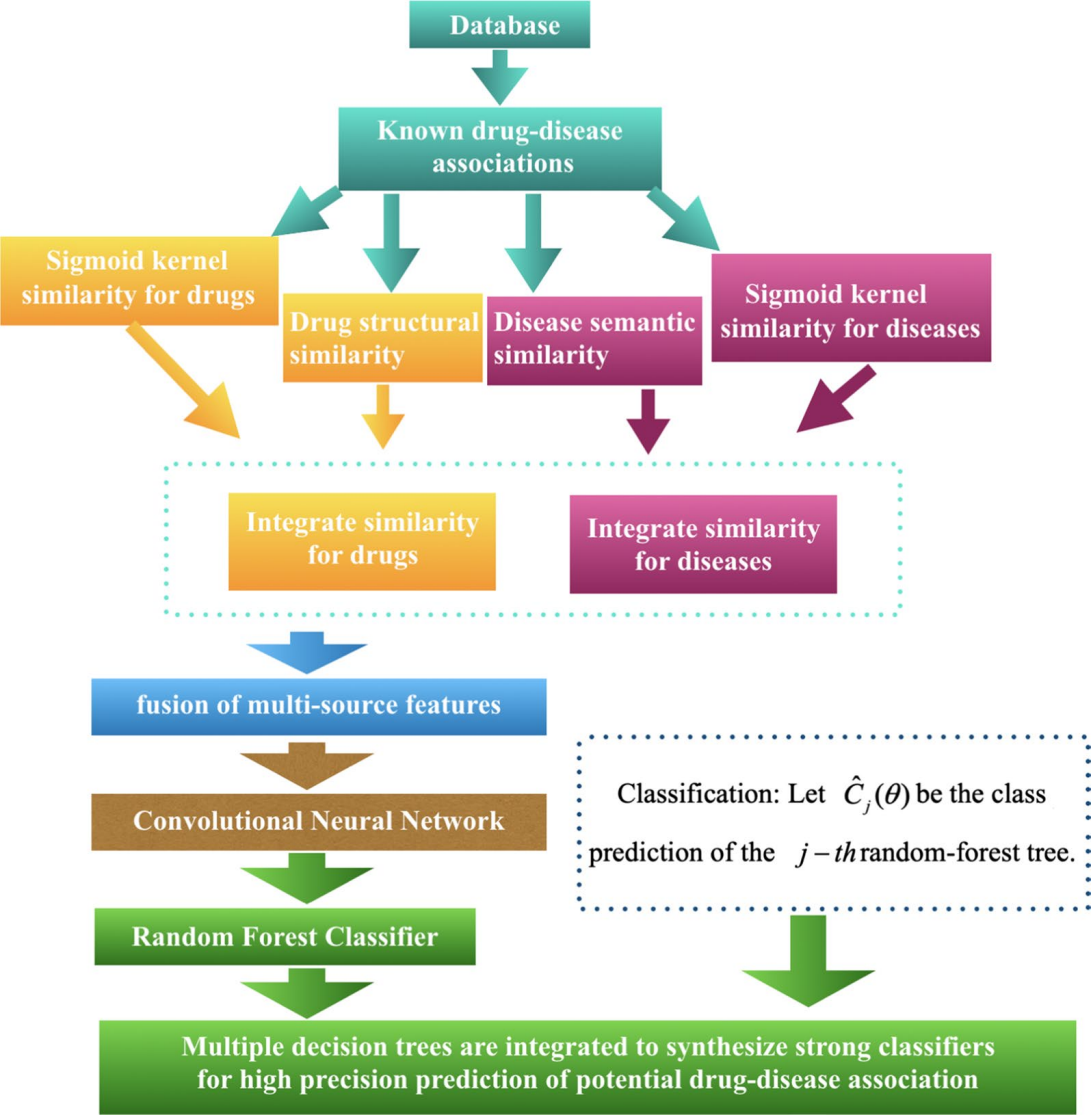
Flowchart of SKCNN model to predict potential drug–disease associations

The flowchart of SKCNN model to predict potential drug–diseases associations is as shown in Fig. 1. SKCNN first calculated the drug sigmoid kernel, disease sigmoid kernel, drug structural similarity and disease semantic similarity respectively. The drug sigmoid kernel is combined with drug structural similarity and disease sigmoid kernel is combined with disease semantic similarity to obtain the drug and disease similar descriptors. It then uses the CNN to extract the features based on the combined drug and disease similarity. In its last step, a random forest classifier is introduced to infer whether the drug–disease pair as the given input is associated or not.

### Datasets

As shown in Table 1, Gottlieb et al. collected 593 drugs, 313 diseases and 1933 validated drug–disease associations from multiple data sources and referred to this data set as the gold standard dataset, which we here abbreviate as Fdataset [19]. In this dataset, the information of drugs is collected from the DrugBank database. Disease information is collected from the Online Mendelian Human Genetics (OMIM) database [20], which focuses on genetic diseases, including textual information and related reference information, sequence records, maps, and other related databases. Luo et al. compiled another dataset called Cdataset which covers 663 drugs, 409 diseases as well as 2532 associations between them [12].

**Table1:** General statistics on Fdataset and Cdataset

Datasets	Drugs	Diseases	Interactions
Cdataset	663	409	2532
Fdataset	593	313	1933

### Similarity for drugs and disease

We here introduce two kinds of drug similarities (drug sigmoid kernel similarity and drug structure similarity) and two kinds of disease similarities (disease sigmoid kernel similarity and disease semantic similarity) in this section. Previous researches show that the sigmoid kernel function which belongs to the global kernel function is effective to extract the global characteristics of the samples. In this work, we used it to extract the features representing each drug–disease association. We construct an adjacency matrix ${\text{A}}$, which briefly store the known and unknown drug–disease association information between drug $d\left( j \right)$ and disease $e\left( i \right)$. The columns of the matrix represent drugs and the rows represent diseases. When drug $d\left( j \right)$ is proved to be related to disease $e\left( i \right)$, elements $A\left( {e\left( i \right),d\left( j \right)} \right)$ are equal to 1, otherwise 0. We defined binary vector $V\left( {d\left( i \right)} \right)$ to represent the association profile of drug $d\left( i \right)$ by observing whether $d\left( i \right)$ is associated with each of disease. The binary vector $V\left( {d\left( i \right)} \right)$ is equivalent to the *i*th column vector of adjacency matrix $A$. The sigmoid kernel for drug $d\left( i \right)$ and drug $d\left( j \right)$ is calculated as follow:

1 $$ Kr\left( {d\left( i \right),d\left( j \right)} \right) = { \tanh }\left[ {a\left( {V\left( {d\left( i \right)} \right) \cdot V\left( {d\left( j \right)} \right)} \right) + r} \right] . $$

where $a$ = $1/N$ and $N$ notes the dimension of the input vector. The value of $r$ is 0.

Similarly, we calculate the sigmoid kernel of the disease, where binary vector $V\left( {e\left( i \right)} \right)$ (or $V\left( {e\left( j \right)} \right)$) represents the interaction profiles of disease $e\left( i \right)$ (or $e\left( j \right)$) by observing whether $e\left( i \right)$ (or $e\left( j \right)$) is associated with each of the drugs and is equivalent to the *i*th (or *j*th) row vector of adjacency matrix $A$. For disease $e\left( i \right)$ and disease $e\left( j \right)$, we calculate the sigmoid nucleus between diseases as follow:

2 $$ Ki\left( {e\left( i \right),e\left( j \right)} \right) = { \tanh }\left[ {b\left( {V\left( {e\left( i \right)} \right) \cdot V\left( {e\left( j \right)} \right)} \right) + z} \right] $$

where $b = 1/M$ and $M$ denotes the dimension of the input similarity. We set the value of $z$ as 0.

Drug structure similarity is calculated based on their chemical structures. We downloaded SMILES from DrugBank [21, 22]. The Chemical Development kit is used to calculate the similarity of two drugs as the Tanimoto score of their fingerprints [23]. The similarity with less predicted information is converted to a value close to zero. The drugs are clustered according to the known relationship between drugs and diseases. We apply the Logistic function to compute the similarity and modify the surface of the genetic-related diseases. The Logistic regression function is defined as follows:

3 $$ L\left( x \right) = \frac{1}{{1 + e^{{\left( {cx + f} \right)}} }} $$

where $x$ denotes the similarity value, c and f are adjusting parameters. Convert small similarity values to values close to zero. At the same time, large similarity values will be enlarged by Logistic function. Then, the drug structure similarity $DE_{r}$ is obtained.

We construct a drug weighted network based on the known drug-disease association. A point in the network is represented by a group of drugs, a group of drugs with a common disease form a edge, and the shared disease of the drug pair represents the weight. As a graphical clustering method, ClusterONE was proposed to the problem of identifying cohesive modules in the field of formaldehyde networks [24]. We here introduced it to identify cluster $C$, which is computed as follows:

4 $$ f\left( C \right) = \frac{{W_{in} \left( C \right)}}{{\left( {W_{in} \left( C \right) + W_{bound} \left( C \right) + P\left( C \right)} \right)}} $$

where $W_{in} \left( C \right)$ denotes the total weight of the inner edges of a set of vertices $C$; $W_{bound} \left( C \right)$ denotes the total weight of the edges connecting the set to the remainder of the group; and $P\left( C \right)$ is the penalty term. We assume that drug $d_{i}$ and drug $d_{j}$ are located in the same cluster $C.$ The drug structure similarity $DE$ between $r_{i}$ and $r_{j}$ is defined as [12]:

5 $$ DE = \left( {1 + f\left( C \right)} \right) * DE_{r} $$

In addition, for the structure similarity between the two drugs, if it is equal to or greater than 1, we use 0.99 instead.

We further calculate another type of disease similarity, that is, disease semantic similarity by using MimMiner, which measures disease similarity by calculating similarities between medical subject words (MeSH) terms [25]. Next, diseases similarity is improved based on the adjusted approaches used in drug structure similarity measure. On this basis, a disease sharing network based on known drug-disease associations was constructed. The points in the network represent diseases, and the weights in the network indicate the number of commonly used drugs for the disease pair. Applying ClusterONE to cluster disease on disease sharing network to enhances the similarity between diseases in the same cluster and obtains a comprehensive disease similarity like drugs. Based on the clustering results, we compute the combined disease similarity $DS$ [12].

### Multi-source feature fusion

In this study, we fuse the different types of disease similarity into one with the sigmoid kernel of the disease, and so do the similarity of drugs. It is anticipated that, using feature fusion can yield more meaningful features that comprehensively reflect the characteristics of the disease and drugs.

For the similarity of drug, we combined drug structural similarity $DE$ and drug sigmoid kernel similarity $Kr$ to form drug similarity $RSim$. The drug similarity $RSim\left( {d\left( i \right),d\left( j \right)} \right)$ for drug $d\left( i \right)$ and drug $d\left( j \right)$ is computed as follow:

6 $$ RSim\left( {d\left( i \right),d\left( j \right)} \right) = \left\{ {\begin{array}{*{20}c} {Kr\left( {d\left( i \right),d\left( j \right)} \right)    if d\left( i \right)\;and\; d\left( j \right)} & {has \;sigmoid \;kernel \;similarity} \\ {DE} & {otherwise} \\ \end{array} } \right., $$

where we use the drug structural similarity $DE$ in the case that the sigmoid kernel of a given drug pair ($d\left( i \right)$ and $d\left( j \right)$) is missing [26].We construct two types of disease similarity, a semantic similarity model $DS$ and a sigmoid kernel similarity $Ki$. The disease similarity $Sim\left( {e\left( i \right),e\left( j \right)} \right)$ for disease $e\left( i \right)$ and disease $e\left( j \right)$ is computed as follows:

7 $$ Sim\left( {e\left( i \right),e\left( j \right)} \right) = \left\{ {\begin{array}{*{20}c} {Ki\left( {e\left( i \right),e\left( j \right)} \right)  if e\left( i \right)\;and\; e\left( j \right)} & {has\; sigmoid \;kernel \;similarity} \\ {DS  \; } & {otherwise} \\ \end{array} } \right.. $$

Similar with the construction of drug similarity, we choose to use disease semantic similarity DS to measure the similarity of a disease pair if their sigmoid kernel similarity is missing.

### Feature extraction based on SKCNN

**Fig. 2:**
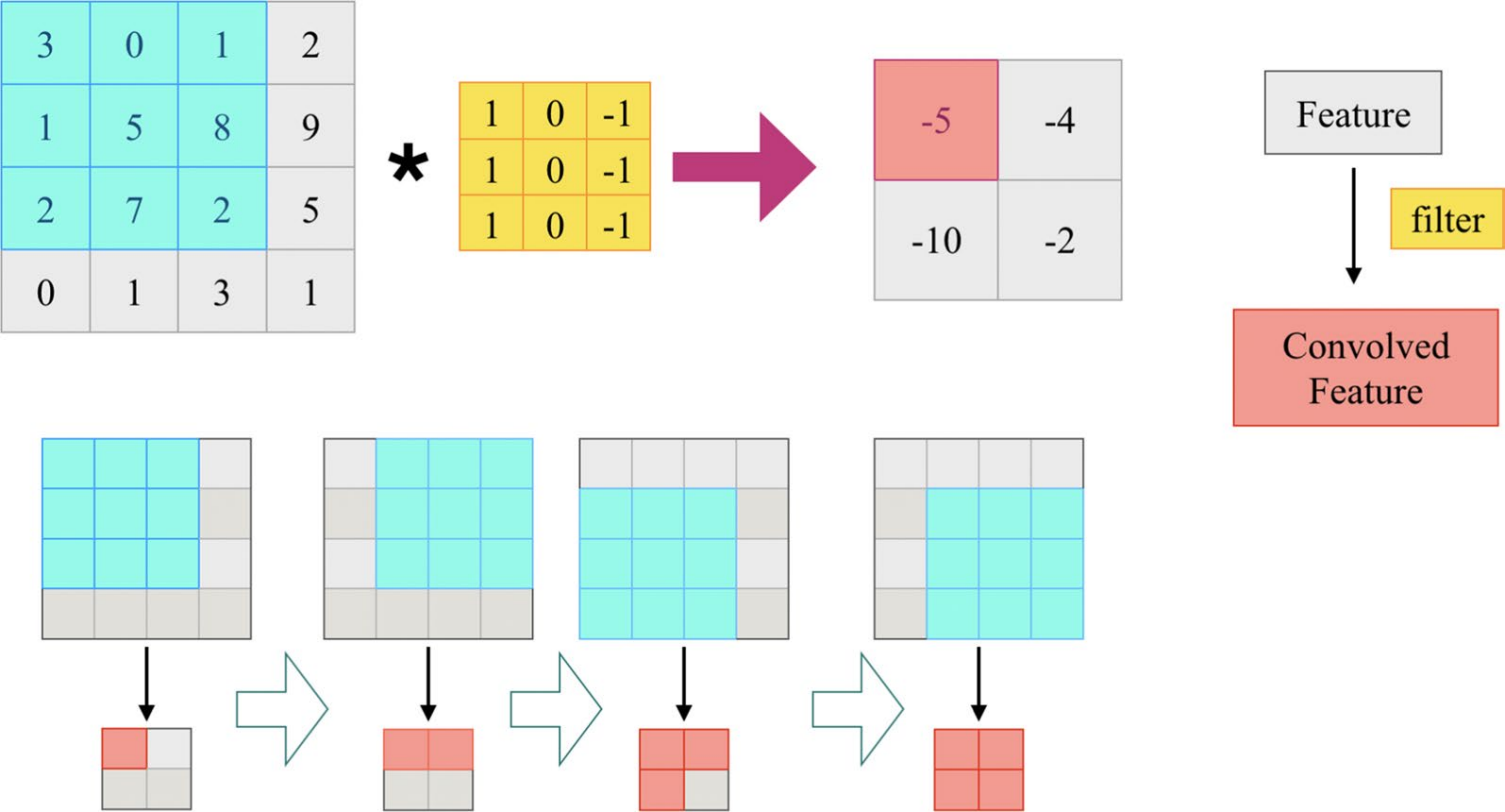
Convolution on features

As an effective solution, deep learning has received extensive attention in the field of bioinformatics. Increasing attention has been attracted by the use of CNN to effectively extract features from different types of raw data, including the type of data we used in this work. We here introduced CNN to further improve the feature representation of drugs and disease in a deep-learning manner. As shown in Fig. 2, we conduct convolution operation on the input similarity using multiple convolution kernels in the convolutional layer.

**Fig. 3:**
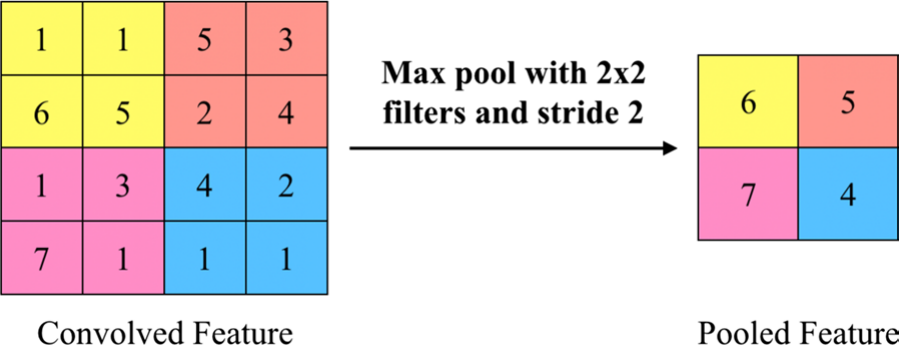
Maximum pooling of features

The mapping process is a forward propagation process, in which the output of the former layer is taken as the input of the latter layer. In *i*th layer, the convolutional operation can be described as:

8 $$ \alpha_{i} = \sigma \left( {\alpha_{i - 1} \otimes W_{i} + b_{i} } \right). $$

where $W_{i}$ denotes the weight matrix of the convolution kernel of *i*th layer; $\otimes$ represents convolution; $b_{i}$ is the offset vector; $\sigma \left( x \right)$ is the activation function. As the next step of convolution, the pooling process is shown in Fig. 3. In the pooling layer $\alpha_{i}$, the pooling is conducted as:

9 $$ \alpha_{i} = subsampling\left( {\alpha_{i - 1} } \right). $$

The Convolutional Neural Network is constructed by using alternate sets of convolutional layers and pooling layers, followed by the feature selection in the pooling layer. Then, the extracted features are learned by the full-connected layer, as well as the probability distribution S. CNN allows the original input matrix $\alpha_{0}$ to be mapped to the new feature expression $S$ by multilevel data transformation or dimension.

10 $$ S\left( i \right) = Map\left( {A = \alpha_{i} |\alpha_{0} ;\left( {W,b} \right)} \right), $$

where $S$ represents the feature expression, $a_{i}$ represents the *i*th label class, and $\alpha_{0}$ represents the original input matrix. The training objective of CNN is to minimize the loss function $F\left( {W,b} \right)$ of the neural network. Meanwhile, the final loss function $E\left( {W,b} \right)$ can be controlled by norm to prevent overfitting, and then the overfitting strength can be controlled by parameter $\lambda$:

11 $$ E\left( {W,b} \right) = F\left( {W,b} \right) + \frac{\lambda }{2}W^{T} W $$

In the training process, the Convolutional Neural Network is optimized by gradient descent method, the parameters of the CNN network are updated layer by layer $\left( {W,b} \right)$, and the learning rate $\eta$ is used to control the intensity of the back-propagation.

12 $$ W_{i} = W_{i} - \eta \frac{{\partial E\left( {W,b} \right)}}{{\partial W_{i} }} $$

13 $$ b_{i} = b_{i} - \eta \frac{{\partial E\left( {W,b} \right)}}{{\partial b_{i} }} $$

In addition, we implemented a series of experiments to optimize the parameters of CNN. As a result, we used a convolution and pooling operation with a kernel size of 16 × 16 for the convolutional layer and 2 × 2 for the subsampling layer. The activation function is set as the sigmoid function; the loss function is set as binary_crossentropy; and Adam is chosen for optimization.

Random forest (RF) is a popular ensemble classifier and is widely used to solve prediction problems, e.g. classification and regression, in different fields including marketing, health insurance and bioinformatics [27]. A multitude of decision trees are constructed in RF for training and the mode of their classification is used to yield the most possible class for input samples. As RF corrects for decision trees’ habit of overfitting to their training set, it generally yields a more stable prediction performance than other types of single classifier such as SVM [28]. As stability and accuracy are of great importance for predicting the association between drugs and diseases on a large scale, in this work, we choose to use RF as the classifier to deal with the features learned by SKCNN.

## Results and discussion

### Evaluation criteria

To evaluate the performance of SKCNN, in this work, we use for types of evaluation criteria to evaluate the performance of the proposed model, i.e., precision (Prec.), F1-score, Recall and accuracy (Acc.).

14 $$ Prec. = \frac{TP}{TP + FP} $$

15 $$ Recall = \frac{TP}{TP + FN} $$

16 $$ F1 - score = \frac{2PR}{P + R} $$

17 $$ Acc. = \frac{TP + TN}{TP + TN + FP + FN}, $$

where TP, FP and FN represent the number of positive samples correctly predicted in the model, the number of correctly predicted negative samples, the number of falsely predicted positive samples and the number of false predicted negative samples, respectively.

### Evaluate prediction performance

To evaluate the performance of SKCNN with regards to the prediction on drug–disease associations, we use tenfold cross-validation on the Fdataset and Cdataset. There are totally 1933 drug–disease associations in Fdataset. In cross validation, we divided original samples into ten disjoint groups, nine of which were selected as training sets each time, and the remaining group was used as a test set, such that we repeat the experiment 10 times. Finally, we yielded the experimental results and computed the mean and standard deviation as the final experimental results for performance evaluation [29]. We performed tenfold cross-validation on two data sets.

We implemented our proposed method on the dataset of Fdataset using tenfold cross validation. Table 2 shows that our proposed model yielded an average accuracy of 89.55%, precision of 91.65%, recall of 87.07% and F1-score of 89.28% with standard deviations of 1.15%, 1.77%, 1.75% and 1.19%, respectively. Table 3 shows that in the experiment on the Cdataset, our method yielded the average accuracy of 91.38%, precision of 92.69%, recall of 89.89%, and F1-score of 91.25% with standard deviations of 1.39%, 1.58%, 2.21% and 1.45%, respectively.

**Table 2:** Experimental results of tenfold cross-validation yielded by SKCNN on Fdataset

Test set	Acc. (%)	Pre. (%)	Recall (%)	F1-score (%)
1	89.69	92.31	86.60	89.36
2	87.37	90.06	84.02	86.93
3	88.66	90.32	86.60	88.42
4	88.86	90.76	86.53	88.59
5	88.86	89.06	88.60	88.83
6	89.64	91.80	87.05	89.36
7	90.93	95.40	86.01	90.46
8	89.38	91.30	87.05	89.12
9	91.45	91.67	91.19	91.43
10	90.67	93.85	87.05	90.32
Average	89.55 ± 1.15	91.65 ± 1.77	87.07 ± 1.75	89.28 ± 1.19

**Table 3:** Experimental results of the tenfold cross-validation yielded by SKCNN on Cdataset

Test set	Acc. (%)	Pre. (%)	Recall (%)	F1-score (%)
1	90.35	92.18	88.19	90.14
2	93.11	95.82	90.16	92.90
3	89.13	90.91	86.96	88.89
4	92.09	95.32	88.54	91.80
5	89.53	91.32	87.35	89.29
6	91.50	91.67	91.30	91.49
7	91.30	91.97	90.51	91.24
8	91.50	93.03	89.72	91.35
9	93.87	93.02	94.86	93.93
10	91.50	91.67	91.30	91.49
Average	91.38 ± 1.39	92.69 ± 1.58	89.89 ± 2.21	91.25 ± 1.45

**Fig. 4:**
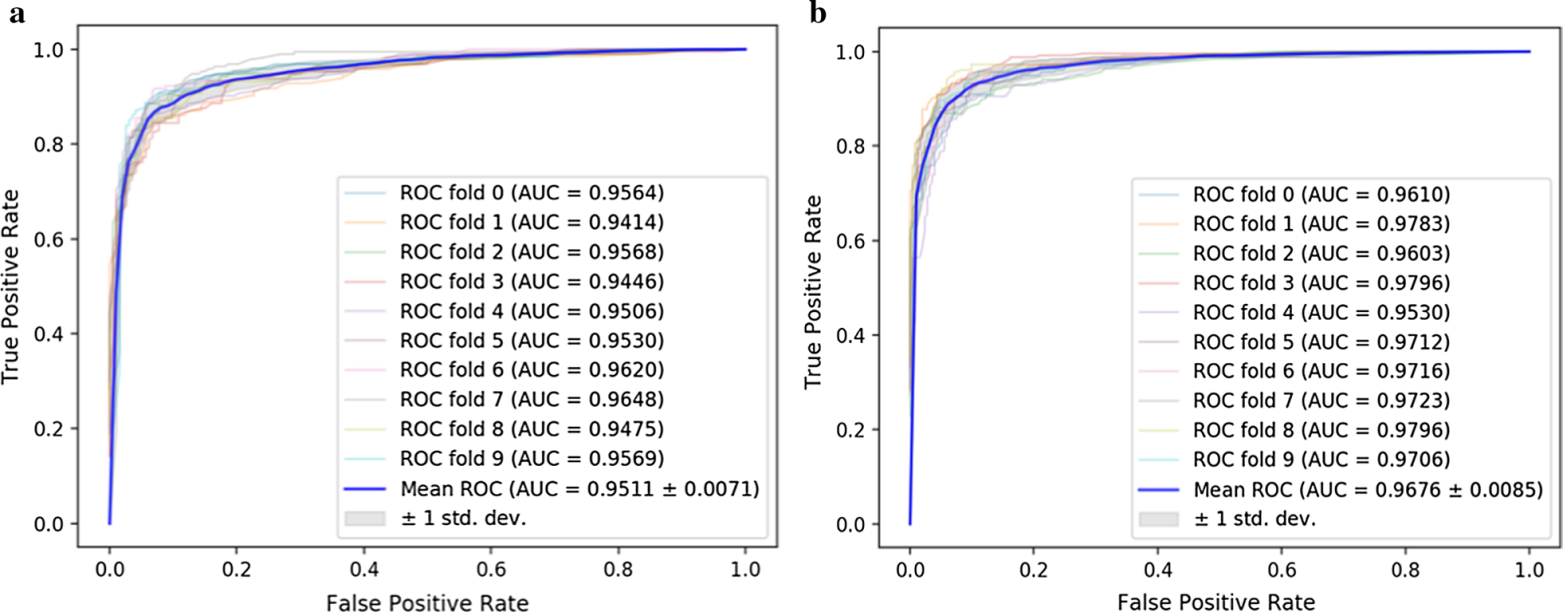
**a**, **b** The ROC curves yielded by SKCNN using tenfold cross validation on the Fdataset and Cdataset, respectively

**Fig. 5:**
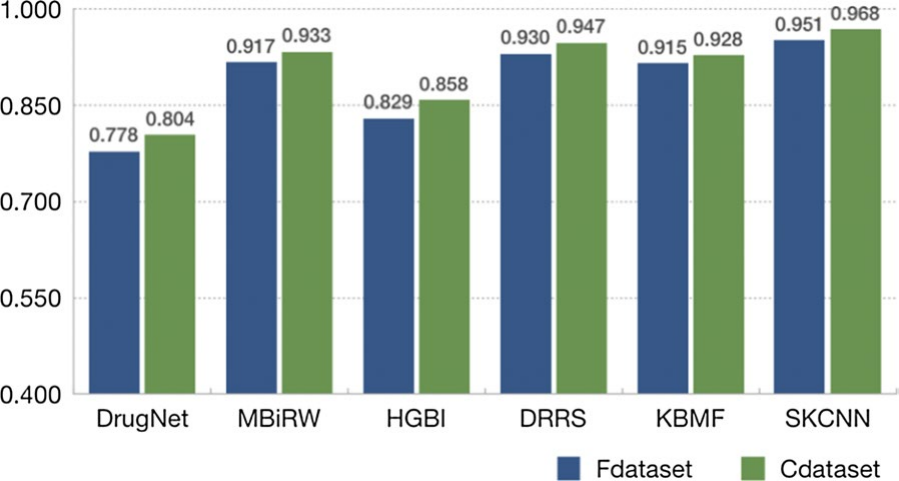
AUC results yielded by different methods using tenfold cross validation

To evaluate the performance of SKCNN, we compare it with five state-of -the-art methods: MBiRW, DrugNet, HGBI, KBMF and DRRs, which are reviewed as aforementioned. The results of SKCNN tenfold cross-validation AUC are shown in Fig. 4. We summarize the experimental results of the six models as shown in Fig. 5. On the Cdataset, SKCNN has an AUC of 0.968. DrugNet, MBiRW, HGBI, KBMF and DRRS yielded poor AUCs of 0.804, 0.933, 0.858, 0.928 and 0.947, respectively. On the Fdataset, SKCNN has an AUC of 0.951. DrugNet has an AUC value of 0.778, MBiRW, HGBI, KBMF and DRRS yielded poor AUC of 0.917, 0.829, 0.915 and 0.930. The results from both two experiments demonstrate that the performance of SKCNN is significantly better than the other five models.

We consider additional statistical analysis should be conducted to quantify how outstanding the prediction performance is compared with the other methods. For this, we performed T test on the AUC values of the six methods on the Fdataset and Cdataset, and the calculated p-values were close to 0.0613 and 0.0534 respectively. Therefore, for the prediction implemented by the six methods on the two datasets, we consider the performance difference significant and anticipate that SKCNN has better performance than the others in real prediction with high possibility.

We also calculated the value of Cohen’s d to measure standardized difference of the prediction performance between the proposed method and the compared ones. As a result, regarding to the AUC values on C dataset, the Cohen’s score was 0.917. For F dataset, the value of Cohen’s d is 0.898. The results show that the effect is significant on two datasets.

### Comparison among different classifier

**Fig. 6:**
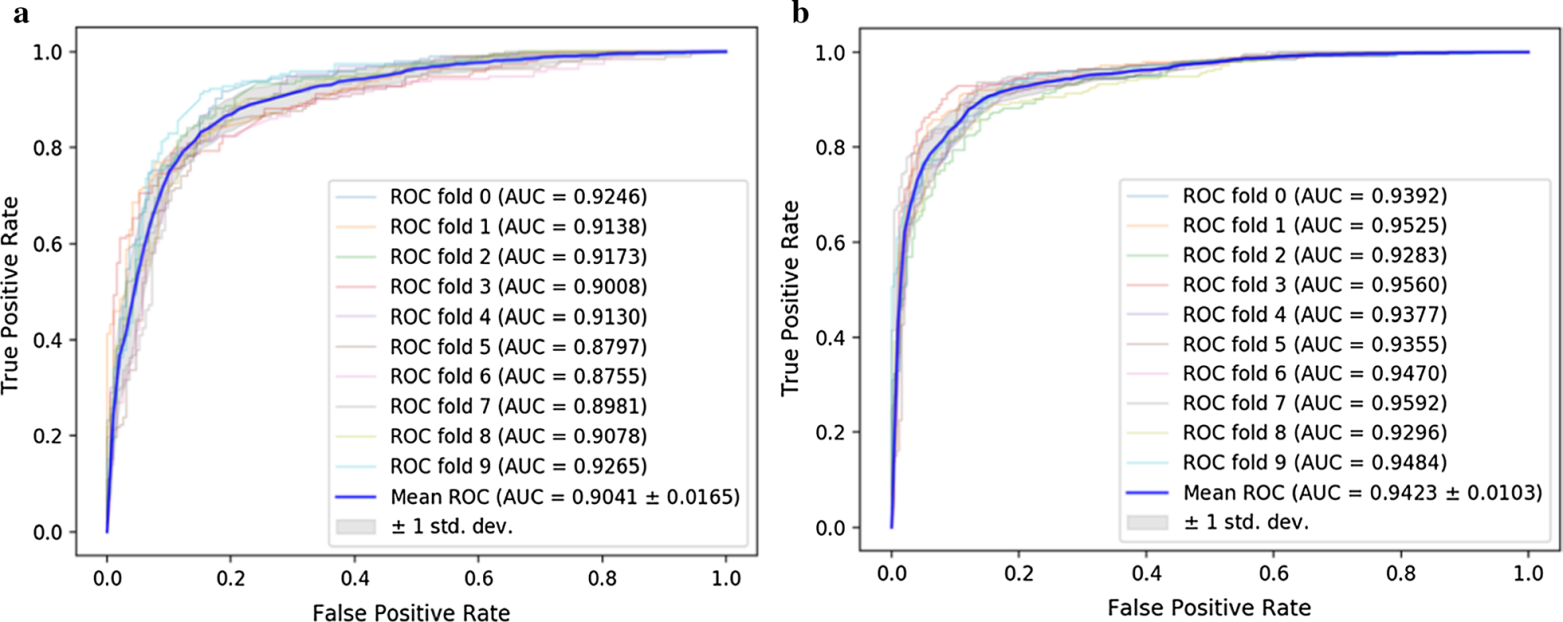
**a**, **b** The ROC curves yielded by SVM using tenfold cross validation on the Fdataset and Cdataset, respectively

To evaluate the performance of random forest that we use to construct our prediction model, we further implemented support vector machine (SVM) classifier on Fdataset and Cdataset using same feature extraction method for performance comparison [30]. SVM is a discriminant classifier defined by the classification hyperplane and widely used to solve classification problems in different domains. Tables 4 and 5 show the results yielded by combining the proposed feature descriptor with support vector machine on Fdataset and Cdataset. In the experiment on Fdataset, SVM yielded an average accuracy of 83.76%, precision of 82.66%, recall of 85.56% and F1-score of 84.02% (see Fig. 6), with standard deviations are 1.54%, 1.98%, 3.61% and 1.70%, respectively. For the prediction on Cdataset, the average accuracy, precision, recall and F1-score are 87.04%, 89.57%, 83.85%, and 86.60% (see Fig. 6), respectively with standard deviations of 1.66%, 1.24%, 2.63% and 1.83%. On the Fdataset, the mean AUC is 0.9041. In the Cdataset, the mean AUC was 0.9423. The performance of both datasets was worse than that of SKCNN.

**Table 4:** Results yielded by SVM on Fdataset using tenfold cross validation

Test set	Acc. (%)	Pre. (%)	Recall (%)	F1-score (%)
1	86.08	83.33	90.21	86.63
2	83.51	83.51	83.51	83.51
3	84.54	81.31	89.69	85.29
4	81.35	82.70	79.27	80.95
5	82.64	82.14	83.42	82.78
6	84.20	84.38	83.94	84.16
7	82.90	83.96	81.35	82.63
8	82.38	78.28	89.64	83.57
9	83.42	81.16	87.05	84.00
10	86.53	85.79	87.56	86.67
Average	83.76 ± 1.54	82.66 ± 1.98	85.56 ± 3.61	84.02 ± 1.70
SKCNN	89.55 ± 1.15	91.65 ± 1.77	87.07 ± 1.75	89.28 ± 1.19

**Table 5:** Results yielded by SVM on Cdataset using tenfold cross validation

Test set	Acc. (%)	Pre. (%)	Recall. (%)	F1-score. (%)
1	86.61	88.43	84.25	86.29
2	89.17	90.95	87.01	88.93
3	84.39	87.50	80.24	83.71
4	90.51	92.18	88.54	90.32
5	85.77	89.52	81.03	85.06
6	86.17	89.96	81.42	85.48
7	87.15	89.50	84.19	86.76
8	87.35	89.54	84.58	86.99
9	85.97	89.57	81.42	85.30
10	87.35	88.57	85.77	87.15
Average	87.04 ± 1.66	89.57 ± 1.24	83.85 ± 2.63	86.60 ± 1.83
SKCNN	91.38 ± 1.39	92.69 ± 1.58	89.89 ± 2.21	91.25 ± 1.45

### Case study

In this section, we selected two diseases, obesity and asthma, for case studies. In the experiments of this section, all known drug–disease associations in the Fdataset are used as training samples of SKCNN. It is worth noting that when predicting the relevance of a particular disease, all associations between a particular disease and the drug should be removed from the training set. Based on the predicted results yielded by SKCNN, we pick up top-20 drugs for confirmation using CTD databases.

Obesity is abnormal or excessive accumulation of fat that poses a risk to a person’s health. It is a major risk factor for diabetes, cardiovascular disease and cancer. As shown in Table 6, 15 out of the top 20 predicted drugs are confirmed after we matched the prediction results with the records of the CTD datasets. Another type of disease we focus on for case study is asthma, a complex disease whose concomitant symptom include paroxysmal wheezing, shortness of breath, chest tightness and cough. It shown that there are about 300 million people have asthma in the world and 30 million people have asthma in China. Table 7 list the top-20 drugs predicted by SKCNN to be associated with asthma. After querying the database of CTD, 17 of them are successfully validated. The case studies of both obesity and asthma demonstrate the promising performance of SKCNN to predict the most potential.

**Table 6:** Top-20 drugs predicted by SKCNN to be associated with obesity based on Fdatabase

Index	Drug name	Evidence	Index	Drug name	Evidence
1	Vigabatrin	Confirmed	11	Fluoxymesterone	NA
2	Sumatriptan	Confirmed	12	Disulfiram	Confirmed
3	Sulindac	Confirmed	13	Carteolol	Confirmed
4	Paroxetine	Confirmed	14	Aspirin	Confirmed
5	Ofloxacin	Confirmed	15	Vincristine	Confirmed
6	Mesalazine	Confirmed	16	Triamcinolone	Confirmed
7	Mercaptopurine	NA	17	Terazosin	NA
8	Isoproterenol	Confirmed	18	Sildenafil	Confirmed
9	Hyoscyamine	Confirmed	19	Sertraline	Confirmed
10	Formoterol	NA	20	Salicyclic acid	NA

**Table 7:** Top-20 drugs predicted by SKCNN to be associated with asthma based on Fdatabase

Index	Drug name	Evidence	Index	Drug name	Evidence
1	Methimazole	Confirmed	11	Quinidine	Confirmed
2	Famotidine	Confirmed	12	Quetiapine	Confirmed
3	Clonazepam	Confirmed	13	Pyridoxine	NA
4	Trimethoprim	NA	14	Propranolol	Confirmed
5	Triamcinolone	Confirmed	15	Propafenone	Confirmed
6	Timolol	Confirmed	16	Promethazine	Confirmed
7	Theophylline	Confirmed	17	Procainamide	Confirmed
8	Tetrabenazine	NA	18	Prednisolone	Confirmed
9	Tamoxifen	Confirmed	19	Praziquantel	Confirmed
10	Ropinirole	Confirmed	20	Pravastatin	Confirmed

## Conclusion

Although the problem of predicting drug–disease association is of great importance for drug repositioning and much effort has been made toward this domain, there were still some challenges that needed to be overcome such as low prediction accuracy and complex data fusion for feature extraction. In this study, we propose a novel deep learning-based computational method called SKCNN to predict drug lists that associated with diseases on a large scale. Specifically, SKCNN is deep-learning technique which offers a computational pipeline that combines Sigmoid Kernel and Convolutional Neural Network. It can effectively integrate the data of known drug-disease associations and different type of side information relevant to drugs and disease.

We evaluate our proposed model on two real datasets that collect experimentally-supported data using tenfold cross validation. The experimental results demonstrate that our proposed method is effective to predict drug–disease association on a large scale. In addition, two case studies on obesity and asthma illustrate the outstanding performance of SKCNN to predict potential drug lists that is associated with specific diseases. Considering that the data we used to train our model is still relatively limited in number, we anticipate that the prediction of our model could be further improved by using more large data and other different types of side information in the future.

## Data Availability

The datasets that we collected in this work is freely available on https://github.com/HanJingJiang/SKCNN.

## Abbreviations

SKCNN: Sigmoid Kernel and Convolutional Neural Network
HNBI: based on information flow on the heterogeneous network
DRRs: drug repositioning recommendation system
LRSSL: Laplacian regularized sparse subspace learning
SCMFDD: a similarity constrained matrix factorization method for the drug–disease association prediction
SMILE: Simplified Molecular Input Line Entry Specification
CNN: convolutional neural network
MBiRW: utilizes some comprehensive similarity measures and Bi-Random walk
HGBI: heterogeneous graph-based inference
KBMF: genomic kernels using Bayesian matrix factorization
AUC: area under the curve
CTD: comparative toxicogenomics database
OMIM: Online Mendelian Inheritance In Man
MeSH: medical subject words
ROC: receiver operating characteristic
RF: random forest
SVM: support vector machine
